# Molecular beam epitaxy growth of peak wavelength-controlled InGaAs/AlGaAs quantum wells for 4.3-μm mid-wavelength infrared detection

**DOI:** 10.1186/1556-276X-8-310

**Published:** 2013-07-03

**Authors:** Zhenwu Shi, Lu Wang, Honglou Zhen, Wenxin Wang, Hong Chen

**Affiliations:** 1 Key Laboratory for Renewable Energy, Beijing Key Laboratory for New Energy Materials and Devices, National Laboratory for Condensed Matter Physics, Institute of Physics, Chinese Academy of Sciences, Beijing, China; 2 State Key Laboratory of Infrared Physics, Shanghai Institute of Technical Physics, Chinese Academy of Sciences, Shanghai 200083, People’s Republic of China

**Keywords:** Molecular beam epitaxy, Quantum well infrared detector, InGaAs/AlGaAs quantum well

## Abstract

InGaAs/AlGaAs multiple quantum wells used for 4.3 μm mid-wavelength infrared quantum well infrared detectors were grown by molecular beam epitaxy. In composition loss was observed and quantitatively studied by high-resolution X-ray diffraction technology. By this In composition loss effect, the energy band engineering on the photo-response wavelength is not easily achieved. A thin AlGaAs barrier grown at low temperature is used to suppress the In atom desorption, and this growth process was verified to be able to adjust the photo-response wavelength as designed by energy band engineering in the photocurrent spectrum.

## Background

Infrared detector technology is one of most important opto-electric devices. It has been developed from bulk material to the quantum well structure [1,2]. The 3 ~ 5 μm middle-wavelength-infrared (MWIR) region is of particular interest in the fields of scientific research, aerial reconnaissance, and missile tracking. The dominant detector in this wavelength field is still HgCdTe (MCT) due to its high quantum efficiency and lower thermal generation rate. However, due to the high density of defect in the MCT material, it is difficult to reduce the dark current of the MCT device [3]. The quantum well infrared photodetector (QWIP) is fabricated from a GaAs-based material, which is expected to have lower dark current due to the mature process on both the material and device for GaAs [4,5].

GaAs-based InGaAs/AlGaAs QWIP working in the MWIR region is studied [6-9]. Low dark current of a few pA was measured in MWIR QWIP based on InGaAs/AlGaAs-strained quantum well grown on GaAs substrates [10]. Recently, a 28% quantum efficiency and a detectivity D^*^ = 7 × 10^11^ Jones at 77 K were reported in a InGaAs/AlGaAs QWIP working below 4.1 μm [11].

Nevertheless, the huge difference of the growth window for the InGaAs and AlGaAs materials and the easy desorption of the In atom make such multiple quantum well system hard to fabricate [12]. Generally, the typical growth temperature of AlGaAs barrier should be higher than 600°C, and the In atom in the InGaAs quantum well starts the desorption at around 520°C [13-15]. This intrinsic property makes the In composition in the InGaAs quantum well quite unstable when increasing the temperature to grow the followed AlGaAs barrier. Besides, the high mobility of In atoms at the substrate made the surface morphology of InGaAs layer very sensitive to the growth parameters [16]. These problems would make the precise peak wavelength control difficult since the absorption peak wavelength is very sensitive to the structural characteristics of QWIP, such as the In composition and its profiles in the quantum well [17,18]. It is important to control the In composition profiles in the InGaAs/AlGaAs quantum well structure growth for optimizing the MIRW-QWIP device.

In this work, the In desorption behavior in the molecular-beam-epitaxy (MBE) growth of InGaAs/AlGaAs MWIR QWIP was studied. With low-temperature capping technology of a thin AlGaAs, the In composition can be well controlled.

## Methods

The samples in this work were grown on a Si-GaAs substrate by a VG-80H MBE system and divided into two groups assigned as groups I and II. The growth rates were firstly determined by reflection high-energy electron diffraction and finely calibrated by X-ray diffraction (XRD) and photoluminescence (PL) measurement.

**Figure 1:**
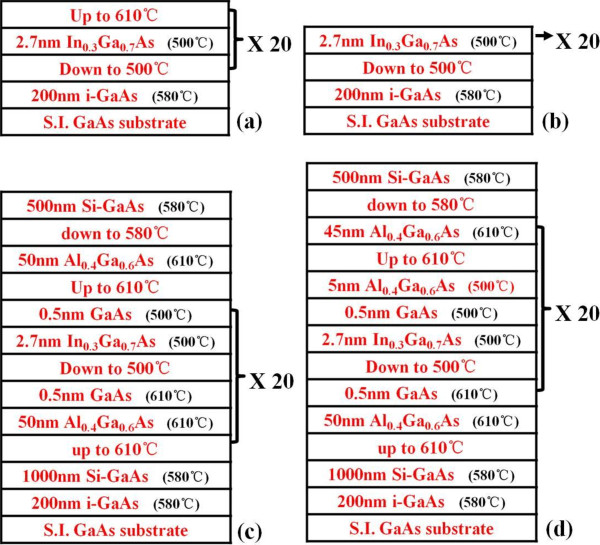
Procedure schematics of (a) sample A, (b) sample B, (c) sample D, and (d) sample E.

Group I including samples A, B, and C were used for observing the In composition-losing behavior in InGaAs. Figure 1a,b shows the growth procedure illustrations of samples A and B, respectively. Sample C was a complete replica of sample A.

In order to demonstrate the effect of a low-temperature thin AlGaAs capping layer on suppressing the In desorption, group II including samples D, E, and F were grown. All the three samples were designed to have the same structure with a peak absorption wavelength from the inter-subband transition in InGaAs/AlGaAs quantum wells around 4.3 μm. The layer sequence of the sample structure is 1 μm Si-GaAs bottom contact layer, 20 periods of quantum wells consisting of 50 nm Al_0.4_Ga_0.6_As barrier, 0.5 nm GaAs, 2.7 nm In_0.3_Ga_0.7_As, and 0.5 nm GaAs. Then, 500 nm Si-GaAs top contact layer was grown to finish the structure. The growing details of samples D and E were displayed in Figure 1c,d, respectively. Finally, we prepared another sample F using the same growth procedure of sample E.

## Results and discussions

Considering the obvious growth temperature difference between InGaAs and AlGaAs, it is wise to grow InGaAs quantum well under low temperature, then increase the growth temperature to grow the AlGaAs barrier in order to achieve best crystal quality. It is without a doubt that such growth procedure will decrease the In composition in the InGaAs quantum well. However, if the In atom desorption behavior is predictable and repeatable, it is still possible to grow a determined In composition quantum well through the growth of an InGaAs layer with higher In composition to compensate the In loss during the increase of substrate temperature. So, we firstly design an experiment to study such phenomenon.

Because both the quantum well composition and thickness contribute to the inter-band transition energy, it is impossible to determine the structural characteristics of the quantum well by PL directly. Besides, the InGaAs quantum well is as thin as around 2.7 nm in the 4.3 μm QWIP, it is also very hard to measure the precise composition by XRD technology. In order to quantitatively explore the In composition-losing behavior in the quantum well, samples A and B were grown. For sample A, 2.7-nm-thin InGaAs layer was grown at 500°C, then the substrate temperature was increased to 610°C to simulate the In desorption behavior during the growth of the InGaAs/AlGaAs quantum wells. Afterwards, the growth temperature was quickly lowered to 500°C. Then, the same growth procedure was repeated 20 times to obtain a nominal 54-nm-thick InGaAs layer for the XRD testing. Meanwhile, for sample B, a 54-nm-thick InGaAs layer was directly grown on the GaAs substrate at 500°C.

As can be easily predicted, the In composition of sample A is lower than sample B. The 30% In composition was measured in sample B, but this value dropped to about 15% in sample A. These results show an average In atom loss of around 50% in the InGaAs quantum well during the growth temperature increase. In order to check the reproducibility of such process, another sample assigned as sample C was grown with identical growth parameter to sample A. However, 17% In composition was obtained this time. According to the intra-band energy calculated by the transfer matrix method, a change of ±2% of around 20% In composition would lead to an absorption peak wavelength shift of around 0.3 μm [17]. Considering the relative narrow absorption peak of QWIP comparing with MCT and other inter-band absorption detectors, such error must be a huge block for the application of the InGaAs/AlGaAs MWIR QWIP in some fields where a precise control in the absorption peak position was required such as MWIR laser detection and CO_2_ monitor.

To further explore the absorption peak control issue of this system serving as middle-wavelength-infrared photodetector, first, sample D was grown using the strategy that the growth temperature was increased to 610°C as soon as the InGaAs well finished for growing the whole AlGaAs barrier. It has already been proven above that such procedure would cause great In composition loss and had no reproducibility. Unlike sample D, another strategy was applied to prepare sample E: after the InGaAs well was grown, a thin 5-nm AlGaAs barrier was pre-deposited at the InGaAs growth temperature (500°C), and then the substrate temperature was quickly risen to 610°C to grow the remaining barrier. At the same time, in order to characterize the reproducibility in peak absorption wavelength of the new strategy, sample F was made a replicate of sample E.

**Figure 2:**
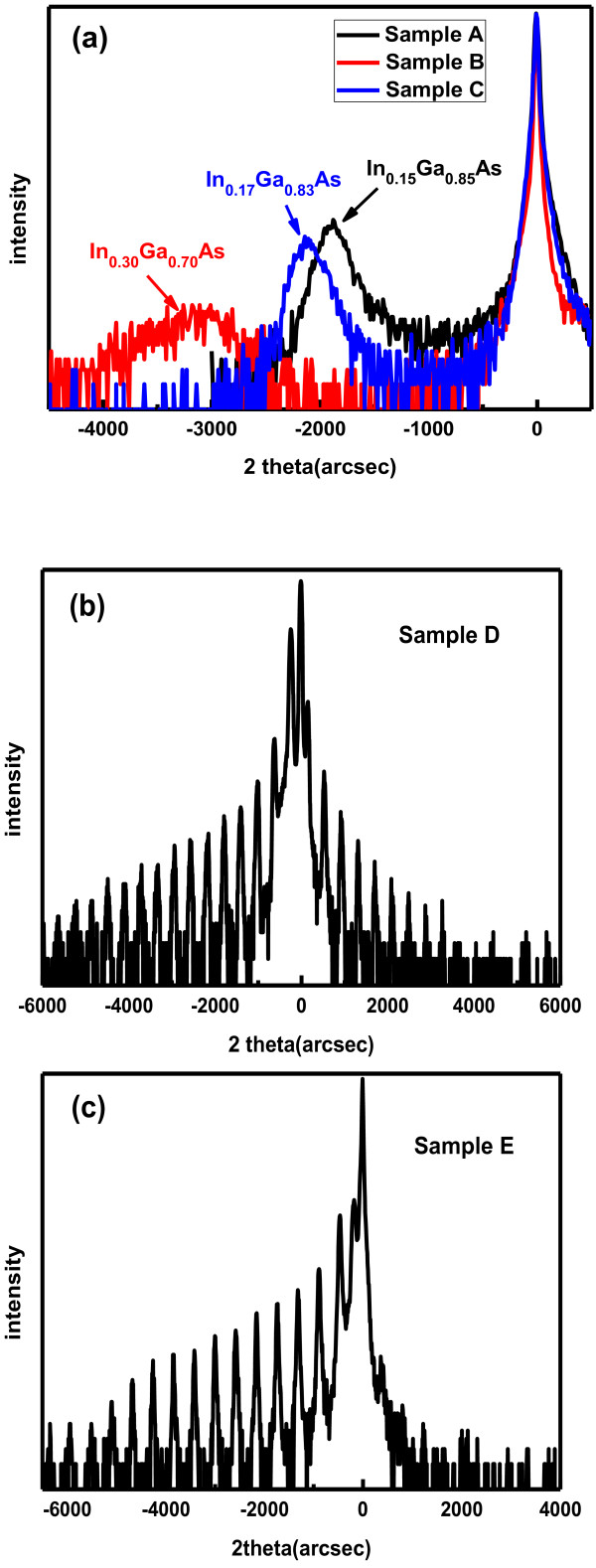
XRD 2 theta-scanning of (a) samples A, B, and C; (b) sample D; (c) sample E.

First, XRD tests were carried out, and Figure 2b,c were the results of samples grown by the two different strategies. In both samples, multiple satellite peaks were observed which show perfect interfacial smoothness. (004) rocking curve measurements showed the full width half maximum (FWHM) of the +1 order satellite: 35 arcsec for sample D and 23 arcsec for sample E. This demonstrated no XRD-sensitive defects during the growth of 5 nm AlGaAs deposited at 500°C. Besides, it was thought that this 5-nm AlGaAs layer grown at low temperature could weaken the effect of strain relaxation and In desorption and segregation when increasing the temperature, and all of these might have contributed to the interface smoothness and InGaAs QW uniformity which both resulted in a narrower FWHM. An interesting phenomenon is that only multiple satellite peaks were observed on the left-hand side of the GaAs substrate peak in the XRD pattern of QWIP with 5-nm low-temperature AlGaAs barrier. However, the satellite peak distribution is nearly symmetrical in the QWIP sample with barrier grown, all at high temperature. We do not have a solid explanation for such phenomenon. It may possibly be related to the higher strain level in the sample containing 5-nm low-temperature AlGaAs barrier [19].

**Figure 3:**
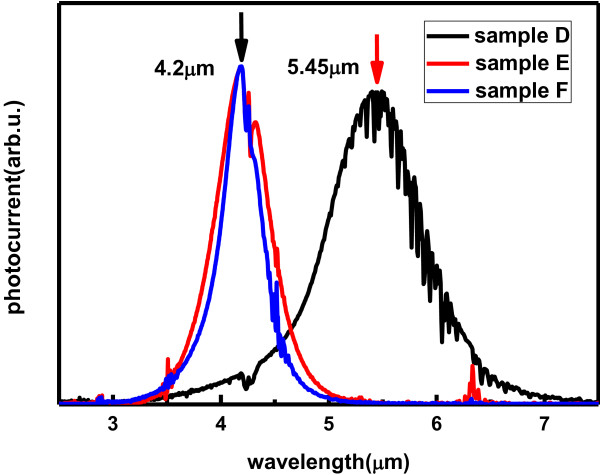
The photocurrent spectrums of samples D, E, and F.

Finally, to evaluate these two strategies in terms of peak absorption wavelength, samples were fabricated into 200 × 200 μm^2^ mesa and then measured by the photocurrent spectrums which were performed by a Fourier transform infrared spectrometer with multi-pass configuration. As can be seen in Figure 3, the peaks of samples E and F were identically located at 4.2 μm well meeting with the theoretic design of around 4.3 μm. However, sample D, without a 5-nm LT-AlGaAs cap layer possessed a wavelength shift of as large as 1.25 μm. According to photocurrent spectrums, the strong photocurrent signal proves the thin LT-AlGaAs barrier does not deteriorate the extraction efficiency very much. So the deposition of thin LT-AlGaAs capping layer is a promising technique to fabricate InGaAs/AlGaAs absorption-wavelength-controlled QWIP, and the stability and reproducibility could be guaranteed as well.

## Conclusion

The In composition loss was found to be a serious problem in the fabrication of InGaAs/AlGaAs QWIP devices due to its unavoidability and unrepeatability. In this study, it was demonstrated that using a thin AlGaAs layer grown at low temperature could successfully prevent the In composition from losing. Highly reproducible peak response wavelengths of InGaAs/AlGaAs QWIP demonstrate the well-controlled structural characteristics of InGaAs quantum well.

## Competing interests

The authors declare that they have no competing interests.

## Authors’ contributions

ZS carried out the sample growth, XRD measurements, and data analysis and drafted the manuscript. LW provided the idea, supervised the study, and co-drafted the manuscript. HZ provided the sample design and conducted the photocurrent spectrum tests. WW and HC coordinated the study. All authors read and approved the final manuscript.
